# Study on the Influence of Image Noise on Monocular Feature-Based Visual SLAM Based on FFDNet

**DOI:** 10.3390/s20174922

**Published:** 2020-08-31

**Authors:** Like Cao, Jie Ling, Xiaohui Xiao

**Affiliations:** Hubei Key Laboratory of Waterjet Theory and New Technology, Wuhan University, Wuhan 430072, China; clk@whu.edu.cn (L.C.); jamesling@whu.edu.cn (J.L.)

**Keywords:** dataset, feature matching, image denoising, visual SLAM, FFDNet

## Abstract

Noise appears in images captured by real cameras. This paper studies the influence of noise on monocular feature-based visual Simultaneous Localization and Mapping (SLAM). First, an open-source synthetic dataset with different noise levels is introduced in this paper. Then the images in the dataset are denoised using the Fast and Flexible Denoising convolutional neural Network (FFDNet); the matching performances of Scale Invariant Feature Transform (SIFT), Speeded Up Robust Features (SURF) and Oriented FAST and Rotated BRIEF (ORB) which are commonly used in feature-based SLAM are analyzed in comparison and the results show that ORB has a higher correct matching rate than that of SIFT and SURF, the denoised images have a higher correct matching rate than noisy images. Next, the Absolute Trajectory Error (ATE) of noisy and denoised sequences are evaluated on ORB-SLAM2 and the results show that the denoised sequences perform better than the noisy sequences at any noise level. Finally, the completely clean sequence in the dataset and the sequences in the KITTI dataset are denoised and compared with the original sequence through comprehensive experiments. For the clean sequence, the Root-Mean-Square Error (RMSE) of ATE after denoising has decreased by 16.75%; for KITTI sequences, 7 out of 10 sequences have lower RMSE than the original sequences. The results show that the denoised image can achieve higher accuracy in the monocular feature-based visual SLAM under certain conditions.

## 1. Introduction

Simultaneous Localization and Mapping (SLAM) has been an important research direction in the field of computer vision and robotics. It is the basic module for many location applications, such as mobile robots, micro aerial vehicles, autonomous driving, virtual reality, augmented reality, and so forth. Various sensors can implement the SLAM algorithm, such as GPS, LiDAR, IMU, and cameras. The camera using image sensors has the advantages of small size, low weight, low power, and low cost, also, it can provide vast information and easy to use. Visual SLAM methods using image sensors have seen tremendous improvements in accuracy, robustness, and efficiency, and have gained increasing popularity over recent years [1]. They can be classified into monocular, binocular, and RGB-D visual SLAM according to the camera used. The monocular visual SLAM system uses only a camera sensor, which is a pure vision issue. In low-cost monocular vision SLAM, image noise as well as its denoising technique is an important issue that needs more investigation.

In visual SLAM, there are three most popular formulations of visual odometry, namely direct, semi-direct, and feature-based methods. In the representative work of these methods, the DSO [2,3] and LSD-SLAM [4,5] are direct methods, the SVO [6,7] is a semi-direct method and the ORB-SLAM [8,9] is a feature-based method. There are lots of problems that have a close influence on the performance of SLAM, such as photometric calibration, motion bias, rolling shutter effect [1], and camera noise. To better understand the influence of noise on the SLAM system, other conditions are fixed as control variables. These methods to deal with the image noise are different. In LSD-SLAM [4,5], it is assumed that the image noise is Gaussian noise, but there is no quantitative analysis of how the noise intensity affects the system. In DSO [2], the study of geometric and photometric noise is performed and the research shows that the DSO modeled by noise is more robust to photometric noise than ORB-SLAM. Because when the photometric noise is high, the feature matching is easy to fail. In the SVO of the semi-direct method and the ORB-SLAM [8,9] of the feature-based method, it is assumed that the feature points are robust to noise, so the noise of the image has not been discussed separately. Image denoising is an effective method in visual SLAM, Cho et al. denoise the image sequences first and improved the visual SLAM performance in a turbid environment [10]. Chen et al. use the DCNNS for semantic segmentation, then the feature matching is limited to the pixel area of the same object in different frames, as a result, the influence of noise on the feature matching is reduced [11]. Liang proposes a precise Iterative Closest Point (ICP) algorithm to overcome noise and outliers to complete precise point cloud registration [12]. Zhang et al. perform feature matching with two past frames to reduce the feature sensitivity to noise [13].

Generally, the visual SLAM methods are based on the assumption that the image has a low noise level. In normal conditions, noise is difficult to notice, even with standard cameras. However, it rises quickly when exposure time has to be long, especially in dark scenes. This is a very challenging task for the existing monocular feature-based SLAM. Existing visual SLAM methods all suffer from accuracy degradation and even failure when the image noise reaches a certain intensity. However, how does image noise affect monocular feature-based visual SLAM, and what will happen to the accuracy and robustness after the image is denoised?

To answer these questions, a dataset containing different noise levels of images is needed. There are many datasets to evaluate the effect of the visual SLAM algorithm at present, such as the TUM dataset [14,15,16], EuRoC dataset [17], KITTI dataset [18,19], and ICL-NUIM RGB-D dataset [20]. However, the images in these datasets are all at low noise levels and have no different levels of noise. Therefore, a dataset for evaluating the noise of the visual SLAM method is extended from the previous publication WHU-RSVI [21]. However, the WHU-RSVI only provides a typical noise level, if the researchers want to study the noise of monocular visual SLAM, different noise levels and types are needed. According to the noise model and clean images introduced in [21], 33 sequences with different noise levels and types are obtained, and the method to add image noise with customized noise types and levels is open-sourced. To reduce the impact of noise on the visual SLAM system, this paper uses the Fast and Flexible Denoising convolutional neural Network (FFDNet) [22] to denoise the image, and then the performance of original sequences and denoised sequences is evaluated.

Scale Invariant Feature Transform (SIFT) [23,24], Speeded Up Robust Features (SURF) [25], and Oriented FAST and Rotated BRIEF (ORB) [26] are the three most commonly used methods in visual SLAM feature extraction and matching. The SIFT description is scale invariance, it can select a correct match of a key point from a large database of other key points, but the calculation is relatively large. SURF is an accelerated robust feature method implemented by integrating images for image convolution. SURF is also a scale and rotation invariant feature description method. ORB improves the problem that the FAST [27,28] detector does not have a direction, and it uses BRIEF [29] to describe the features. Compared with SIFT and SURF, ORB is rotation invariance and resistant to noise and greatly reduces the time required for extracting features.

To evaluate the impact of different levels of noise on matching performance, the three commonly used features were evaluated on the proposed dataset. Then, according to the evaluation, a method using FFDNet to improve ORB-SLAM2 is proposed.

The contributions of this paper are as follows:

(1) An open-source dataset extended from WHU-RSVI [21] for evaluating the noise of Monocular visual SLAM systems or denoising tasks.
(2) An open-source software for adding image noise with customized noise types and levels.
(3) Quantitative evaluation of the influence of different levels of noise on the feature matching of SIFT, SURF, and ORB.
(4) Accuracy improvement of ORB-SLAM2 system due to the image denoising.

All data, documents, programs, and scripts can be used under the Creative Commons Attribution-ShareAlike 3.0 Unported License at http://aric.whu.edu.cn/whu-noise-dataset.html.

To evaluate the influence of noise on the visual SLAM methods, a dataset with different levels of image noise is generated. The previous work of this dataset is WHU-RSVI [21], where the open-source ray-tracing software POV-Ray to generate images is used.

The model of the image is from “office” of “The Persistence of Ignorance” (http://www.ignorancia.org/), and the size of the model is 800×500×250 cm${}^{3}$. The resolution of the images in the dataset is 640×480, and the camera’s field of view is 90 degrees and the intrinsic matrix of the image in the right-handed coordinates can be expressed as

(1) $$K=\left[\begin{array}{ccc} 320.0 & 0.0 & 319.5\\ 0.0 & 320.0 & 239.5\\ 0.0 & 0.0 & 1.0 \end{array}\right].$$

The function that represents the relationship between brightness and irradiance of image pixel values is called the Camera Response Function (CRF), it is a variety of linear and non-linear relationships that cameras are subjected to during imaging. The real CRF is analyzed in detail and a diverse database of real-world camera response functions is collected by Grossberg and Nayar [30]. Linear CRF is a common response model [31] and the CRF is set to linear in this paper.

Unlike images captured by real cameras, the images generated by POV-Ray are noise-free. There are many types of noise in an image, such as photon shot noise, dark current shot noise, offset fixed pattern noise, dark current fixed pattern noise, source follower noise, quantization noise, and sense node reset noise [32]. The noise model is simplified by Liu et al. [33], and it can be expressed as

(2) $$I=f\left(L+n_{s}+n_{c}\right)+n_{q},$$

where *I* is the pixel brightness from 0 to 255, f(·) represents the CRF, $n_{s}$ is all noise components that depend on irradiance, $n_{c}$ denotes the independent noise before correction and $n_{q}$ is the additional quantization noise. Since most cameras can achieve very low quantization noise, $n_{q}$ is ignored in the model of this paper. The expectation of the irradiance-dependent noise is $E\left(n_{s}\right)=0$, and the variance of it is $\mathrm{Var}\left(n_{s}\right)=L\sigma_{s}^{2}$, the expectation of irradiance-independent noise is $E\left(n_{c}\right)=0$, and the variance of it is $\mathrm{Var}\left(n_{c}\right)=\sigma_{c}^{2}$ and *L* represents the irradiance. Grossberg and Nayar [30] normalized the irradiance *L* and the RGB value from 0 to 1, in this paper, the irradiance *L* value and the RGB value are discrete from 0 to 255 as mentioned in [34], $\sigma_{s}$ and $\sigma_{c}$ are also dimensionless values.

**Figure 1:**
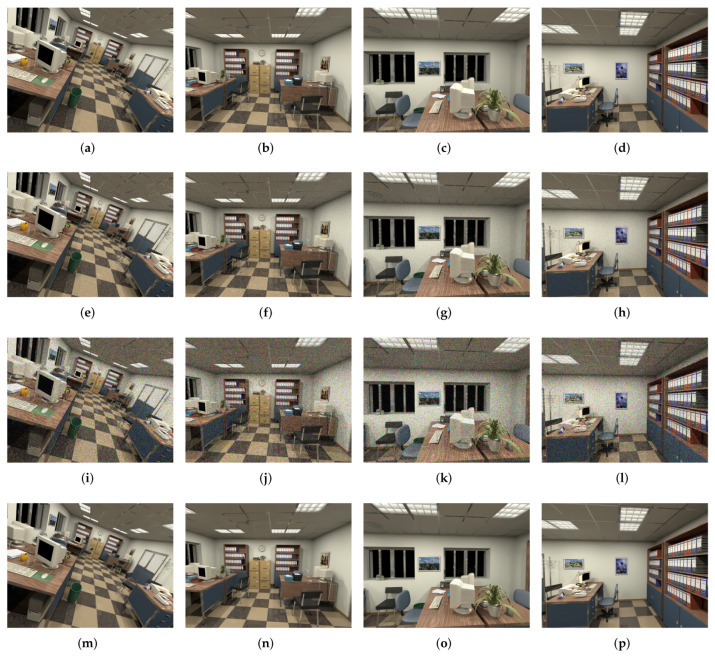
Noise dataset overview. (**a**–**d**) Clean images; (**e**–**h**) Medium noisy images with noise Medium noise intensity $\sigma_{s}=0.06$, $\sigma_{c}=0.03$; (**i**–**l**) High noisy images with noise Medium noise intensity $\sigma_{s}=0.2$, $\sigma_{c}=0.1$; (**m**–**p**) Denoised images for Medium noisy images.

By taking different $\sigma_{s}$ and $\sigma_{c}$, images with different noise levels can be obtained according to the Equation (2). It is assumed that the noise levels are the same on the three channels of R, G, and B and the image noise can be divided into three types: irradiance-independent, irradiance-dependent, and the mixture of the two. Irradiance-independent noise is the so-called Gaussian noise. Noisy images of different levels can be obtained by adjusting $\sigma_{c}$. In this paper, $\sigma_{c}$ ranges from 0.005 to 0.10. Except for the value of $\sigma_{c}$ = 0.005, the $\sigma_{c}$ is incremented by a step size of 0.01. The irradiance-dependent noise is obtained by adjusting $\sigma_{s}$. The value of $\sigma_{c}$ in this paper ranges from 0.01 to 0.20. Except for the value of $\sigma_{s}$ = 0.01, the other steps are 0.02. For the noise mixed by the irradiance-independent and the irradiance-dependent, the value of $\sigma_{s}$ is twice the value of $\sigma_{c}$, and the steps are the same as that when the value is taken separately. The images of different noise levels in the final sequence are shown in Figure 1.

## 2. Methods

### 2.1. Noise Dataset

By modeling the trajectories of the sequence through B-spline curves, the ground truth of the trajectories of each sequence can be obtained. The format of the ground truth in the dataset is compatible with the TUM dataset. All sequences with different noise levels in the dataset have the same ground truth and the statistical information of the sequence is shown in Table 1.

### 2.2. Image Denoising

Image denoising is an important research direction in low-level vision. Noise inevitably occurs during the imaging process, which will seriously affect the quality of the image. Therefore, image denoising is an essential step in many image processing and computer vision tasks.

**Figure 2:**
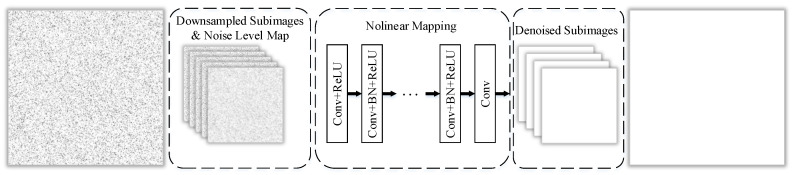
The structure of FFDNet.

FFDNet [22,35] is used to denoise the images, in this paper, the model trained by Zhang et al. [22] using an open-source machine learning framework PyTorch (https://opencv.org/). FFDNet is an image denoising method based on the convolutional neural network developed in recent years. It is proposed based on DnCNN (denoising convolutional neural network) [36]. Compared with existing neural network denoising works DnCNN [36] and BM3D [37], FFDNet has faster execution time and smaller memory footprint, and it can use a single network model to deal with various levels of noise. It has a low calculation and can handle spatial shift noise, as shown in Figure 2.

As shown in Figure 2, the input tensor is then fed into a series of 3 × 3 convolutional layers, each of which contains the following three operations—Conv, ReLU (rectified linear unit), and BN (Batch Normalization) [38]. Specifically, the first layer uses Conv + ReLU, the middle is Conv + BN + ReLU, and the last convolution layer is Conv. After each convolution, zero padding is used to ensure that the size of the feature map does not change. After CNN, the upscaling operation is used as the reverse process of the downsampling process in the input stage to generate a denoised image with the same size as the original input image.

**Figure 3:**
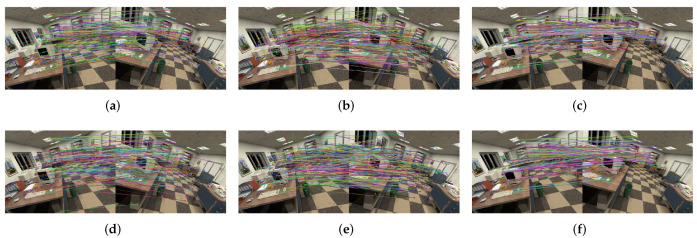
Feature matching overview. (**a**–**c**) Feature matching by SIFT, SURF, and ORB of the noisy images; (**d**–**f**) feature matching by SIFT, SURF and ORB of the denoised images.

In FFDNet, it takes a tunable noise level map as input to make the denoising model flexible to noise levels. To improve the efficiency of the denoiser, a reversible downsampling operator is introduced to reshape the input image of size W×H×C into 4 downsampled sub-images of size $\frac{w}{2}\times \frac{H}{2}\times 4C$, where *W* and *H* are the width and height of the input image, and *C* is the number of channels. For grayscale images, C=1, for color images, C=3. The downsampling process can significantly improve the training speed without reducing the modeling ability. By considering the balance of complexity and performance, it is empirically set the number of convolution layers as 15 for grayscale images and 12 for color images. As to the channels of feature maps, it is set 64 for grayscale images and 96 for color images. Besides, unlike DnCNN, denoising the down-sampled sub-images can effectively increase the receiving domain and thus obtain a suitable network depth. After the downsampling operation, it is fed into the CNN together with the adjustable noise level map, so that images with different noise levels can be processed. The image after image denoising is shown in (m)–(p) of Figure 3.

Zhang et al. [22] evaluated the running time of FFDNet (The evaluation was performed in Matlab (R2015b) environment on a computer with a six-core Intel(R) Core(TM) i7-5820K CPU @ 3.3 GHz, 32 GB of RAM and an Nvidia Titan X Pascal GPU). The authors find that for a gray image with a size of 512 × 512, the denoising time is 0.012 s (at 83.3 Hz). The image size of our dataset is 640 × 480, which is close to 512 × 512 in terms of data size. It indicates that when the computer has a high-performance GPU, FFDNet can meet the real-time requirements of visual SLAM.

The average Peak Signal to Noise Ratio (PSNR) of the noisy sequences and denoised sequences are shown in Table 2.

### 2.3. Feature Matching

The extraction and matching algorithms provided in OpenCV (https://opencv.org/) are used to compare the matching effects of the three features on the noise dataset. SIFT, SURF, and ORB are used to extract nearly 500 features for every image. Both SIFT and ORB can specify the number of extracted feature numbers, where SURF cannot be set to extract fixed and it is obtained according to the Heisen threshold. The same Heisen threshold results in different feature numbers in different images. So the Heisen threshold is adjusted for each image to extract 500 features in this paper.

The brute-force matching is performed after the feature extraction, that is, the distance between the descriptors of the features in the two images is measured, and then sorted, and the closest distance is taken as the matched pair. The distance between the descriptors expresses the similarity between the two features. For SIFT and SURF, the distance is generally expressed using Euclidean distance. For ORB, the Hamming distance is often used as it uses a binary descriptor. The Hamming distance between two strings of equal length is the number of positions where the corresponding symbols are different.

The ground truth is used to verify the quality of feature matching. Depth of the images is obtained by MegaPOV (http://megapov.inetart.net/), and the projection model of the pinhole camera can be expressed as

(3) $$\begin{array}{cc} d_{1}\left(\begin{array}{c} u_{1}\\ v_{1}\\ 1 \end{array}\right)= & K\left(R_{1}P+t_{1}\right)=\left[\begin{array}{ccc} f_{x} & 0 & c_{x}\\ 0 & f_{y} & c_{y}\\ 0 & 0 & 1 \end{array}\right]\left[\begin{array}{c} R_{1_{00}}x+R_{1_{01}}y+R_{1_{02}}z+t_{1_{0}}\\ R_{1_{10}}x+R_{1_{11}}y+R_{1_{12}}z+t_{1_{1}}\\ R_{1_{20}}x+R_{1_{21}}y+R_{1_{22}}z+t_{1_{2}} \end{array}\right]\\ = & \left[\begin{array}{ccc} (f_{x}R_{1_{00}}+c_{x}R_{1_{20}})x & (f_{x}R_{1_{01}}+c_{x}R_{1_{21}})y & \left(f_{x}R_{1_{02}}+c_{x}R_{1_{22}}\right)z+f_{x}t_{1_{0}}+c_{x}t_{1_{2}}\\ (f_{y}R_{1_{10}}+c_{y}R_{1_{20}})x & (f_{y}R_{1_{11}}+c_{y}R_{1_{21}})y & \left(f_{y}R_{1_{12}}+c_{y}R_{1_{22}}\right)z+f_{y}t_{1_{1}}+c_{y}t_{1_{2}}\\ R_{1_{20}}x & R_{1_{11}}y & R_{1_{22}}z+t_{1_{2}} \end{array}\right], \end{array}$$

where K is the camera’s intrinsic matrix, $P={\left(x,y,z\right)}^{T}$ is the point in the space, $R_{1}$ is the rotation matrix of the first image, $t_{1}$ is the translation vector and $d_{1}$ is the depth of the pixel. According to the Cramer’s rule, the linear equations can be solved, we define that

(4) $$D=\left|\begin{array}{ccc} f_{x}R_{1_{00}}+c_{x}R_{20} & f_{x}R_{1_{01}}+c_{x}R_{1_{21}} & f_{x}R_{1_{02}}+c_{x}R_{1_{22}}\\ f_{y}R_{1_{10}}+c_{y}R_{20} & f_{y}R_{1_{11}}+c_{y}R_{1_{21}} & f_{y}R_{1_{12}}+c_{y}R_{1_{22}}\\ R_{1_{20}} & R_{1_{11}} & R_{1_{22}} \end{array}\right|,$$

(5) $$D_{x}=\left|\begin{array}{ccc} ud_{1}-f_{x}t_{1_{0}}-c_{x}t_{1_{2}} & f_{x}R_{1_{01}}+c_{x}R_{1_{21}} & f_{x}R_{1_{02}}+c_{x}R_{1_{22}}\\ vd_{1}-f_{y}t_{1_{1}}-c_{y}t_{1_{2}} & f_{y}R_{1_{11}}+c_{y}R_{1_{21}} & f_{y}R_{1_{12}}+c_{y}R_{1_{22}}\\ d_{1}-t_{1_{2}} & R_{1_{11}} & R_{1_{22}} \end{array}\right|,$$

(6) $$D_{y}=\left|\begin{array}{ccc} f_{x}R_{1_{00}}+c_{x}R_{1_{20}} & ud-f_{x}t_{1_{0}}-c_{x}t_{1_{2}} & f_{x}R_{1_{02}}+c_{x}R_{1_{22}}\\ f_{y}R_{1_{10}}+c_{y}R_{1_{20}} & vd-f_{y}t_{1_{1}}-c_{y}t_{1_{2}} & f_{y}R_{1_{12}}+c_{y}R_{1_{22}}\\ R_{1_{20}} & d_{1}-t_{1_{2}} & R_{1_{22}} \end{array}\right|,$$

(7) $$D_{z}=\left|\begin{array}{ccc} f_{x}R_{1_{00}}+c_{x}R_{1_{20}} & f_{x}R_{1_{01}}+c_{x}R_{1_{21}} & ud_{1}-f_{x}t_{1_{0}}-c_{x}t_{1_{2}}\\ f_{y}R_{1_{10}}+c_{y}R_{1_{20}} & f_{y}R_{1_{11}}+c_{y}R_{1_{21}} & vd_{1}-f_{y}t_{1_{1}}-c_{y}t_{1_{2}}\\ R_{1_{20}} & R_{1_{11}} & d_{1}-t_{1_{2}} \end{array}\right|,$$

where *D* is a nonzero determinant, then *x*, *y* and *z* in the equations have a unique solution, whose individual values for the unknowns are given by:

(8) $$x=\frac{D_{x}}{D},\;y=\frac{D_{y}}{D},\;z=\frac{D_{z}}{D}$$

According to the *x*, *y*, *z* and the known $d_{2}$, $R_{2}$ and $t_{2}$, the pixel of the point *P* in the second image can be caculated by:

(9) $$d_{2}\left(\begin{array}{c} u_{2}\\ v_{2}\\ 1 \end{array}\right)=K\left(R_{2}\left(\begin{array}{c} x\\ y\\ z \end{array}\right)+t_{2}\right).$$

The error between the matched points is calculated and it can be expressed as

(10) $$e_{match}={\left(u_{2}-u_{1}\right)}^{2}+{\left(v_{2}-v_{1}\right)}^{2}.$$

It is assumed that when the error is within 4 pixels, the match is correct. The point that matches correctly is determined as the inliers, otherwise, it is determined as the outliers.

## 3. Results and Discussions

The experiment platform of this paper is Intel Core i7-7700HQ (4 cores @ 2.80 GHz), 16 GB memory, and NVIDIA GeForce GTX 1050Ti. The mean execution time of FFDNet on the proposed dataset is 0.153 s per frame, and the tracking time of the ORB-SLAM is 0.025 s per frame.

### 3.1. Results of Feature Matching

Multiple pairs of images from different scenes in the dataset are selected for image matching, and the statistical analysis on irradiance-independent noise, irradiance-dependent noise, and mixed noise of the two is performed.

**Figure 4:**
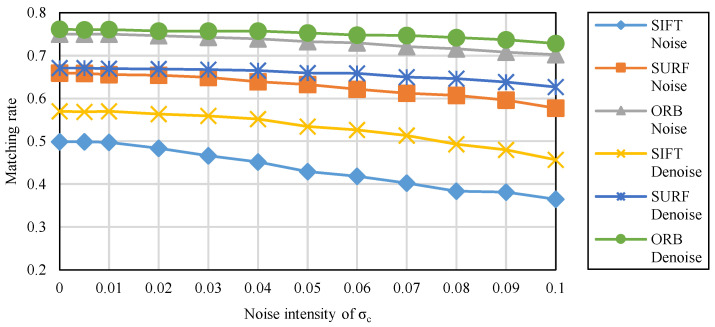
The matching rate of feature matching under the irradiance-independent noise.

The results of irradiance-independent Gaussian noise are shown in Figure 4. It can be seen that the matching rate of ORB is the highest of the three, while SURF is second and SIFT is the lowest.

**Figure 5:**
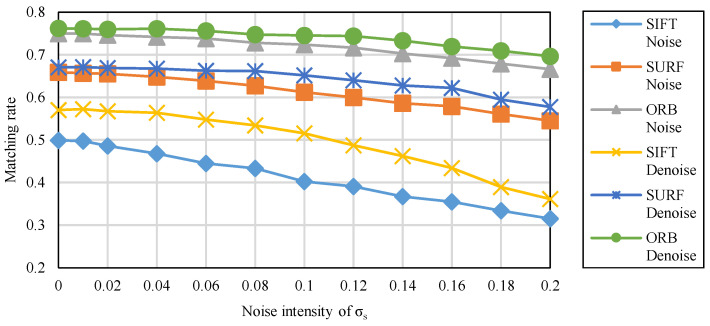
The matching rate of feature matching under the irradiance-dependent noise.

**Figure 6:**
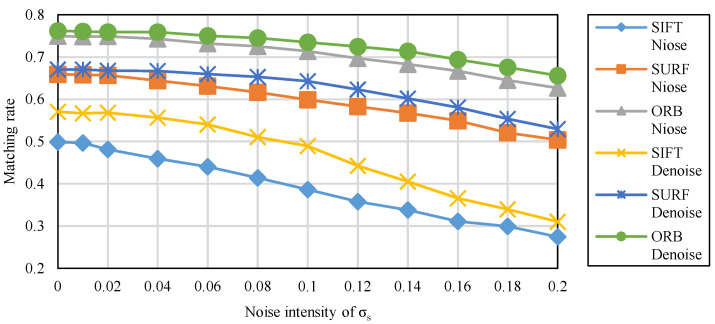
The matching rate of feature matching under the mixed noise.

The results of irradiance-dependent noise and the mixture of the two are shown in Figure 5 and Figure 6. Similar to Gaussian noise, irradiance-dependent noise and the mixed noise of the two are also ORB with the highest matching rate. Different from Gaussian noise, as the intensity of noise increases, the matching rate of irradiance-dependent noise and mixed noise matching image pairs decreases more significantly. For the denoised image, its matching rate is higher than the original image whether it is on ORB, SURF, or SIFT.

The results of feature matching that performed on the original images and the denoised images are shown in Table 3. It is shown that for the denoised image, a higher correct matching rate can be achieved.

### 3.2. Results of Trajectories

The ORB-SLAM2 [9] is used to evaluate the dataset sequences. 1000 features are extracted from each image, the scale factor for the image pyramids is set to 1.2, and the pyramid level is set to 8. The image pyramid is to downsample the image at different levels to obtain images with different resolutions.

The three types of image sequences: irradiance-independent noise, irradiance-dependent noise, and mixed noise are evaluated and compared with the sequence after image denoising.

#### 3.2.1. Noised Sequences

For the noisy sequences in the dataset, each sequence is evaluated for 10 times, and the mean values after excluding outliers are used to represent the evaluation results. The irradiance-independent noisy sequences can be successfully tracked when $\sigma_{c}$ is below 0.05, while the tracking failure occurs when $\sigma_{c}$ is greater than 0.05. As the noise intensity continues to increase, the RMSE of ATE increases significantly. From the noise intensity $\sigma_{c}=0.005$ to $\sigma_{c}=0.05$, the RMSE of ATE increased from 6.7 cm to 26.2 cm with an increase of 19.5 cm. For the denoised images, all sequences in this dataset can be successfully tracked, and the ATE is maintained at about 6.6 cm. This can also reflect that FFDNet has a good effect on irradiance-independent Gaussian noise. The evaluation results are shown in Table 4.

For irradiance-dependent noise, it is found that when the noise intensity $\sigma_{s}<0.04$, the noisy sequences can keep the ATE stable. As the noise intensity continues to increase, the RMSE of ATE increases significantly. When $\sigma_{s}=0.01$, the RMSE of ATE is 7.3 cm, and when $\sigma_{s}=0.08$, it increases to 27.5 cm, with an increase of 20.2 cm. For the denoised sequences, all can be successfully tracked before the noise intensity $\sigma_{s}=0.16$. And the RMSE is maintained stable during this period as shown in Table 4.

The mixed noisy sequences are evaluated after the irradiance-independent noisy sequences and the irradiance-dependent sequences. The results are shown in Table 4. It is found that the tracking failure occurs when the noise intensity is higher than $\sigma_{s}=0.06,\sigma_{c}=0.03$.

The RMSE of ATE of the sequences are 7.1 cm at the noise intensity $\sigma_{s}=0.01,\sigma_{c}=0.005$, and it increases to 14.8 cm when the noise intensity is $\sigma_{s}=0.04,\sigma_{c}=0.02$, which indicates that the mixed noise has the largest effect on the robustness of ORB-SLAM2.

The evaluation results show that the denoised sequences have significantly improved robustness. In terms of accuracy, no matter the noise level, the accuracy of the denoised sequences is higher than that of the noisy sequences.

#### 3.2.2. Clean Sequence

**Figure 7:**
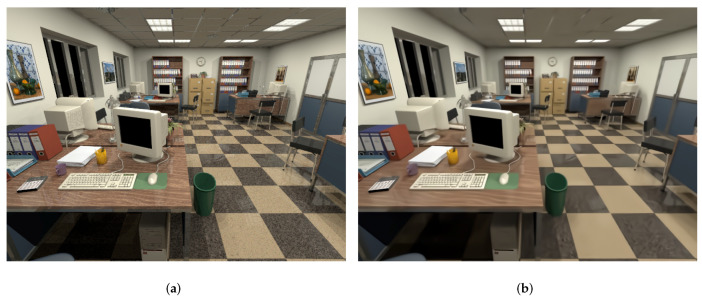
(**a**) Example of the original clean image. (**b**) Example of the clean image after denoising.

As shown in Figure 7, the clean images (noise intensity is zero) are different from the clean images after denoising. Generally, the denoised image looks smoother and softer because the noise level of an image is estimated by FFDNet, some parts that are not noise will be treated as noise and removed. As a result, the clean images after denoising by FFDNet are not the same as the original clean images.

The clean sequence is evaluated separately for 20 times and the results of the evaluation are shown in Table 5.

**Figure 8:**
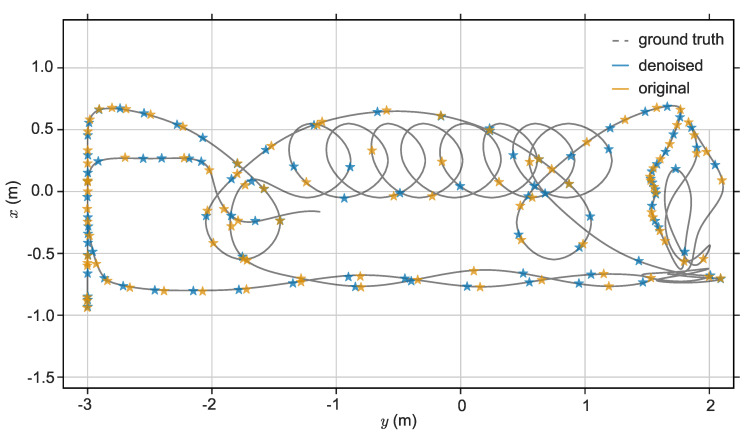
The estimated trajectory of the clean sequence, denoised clean sequence, and their ground truth.

**Figure 9:**
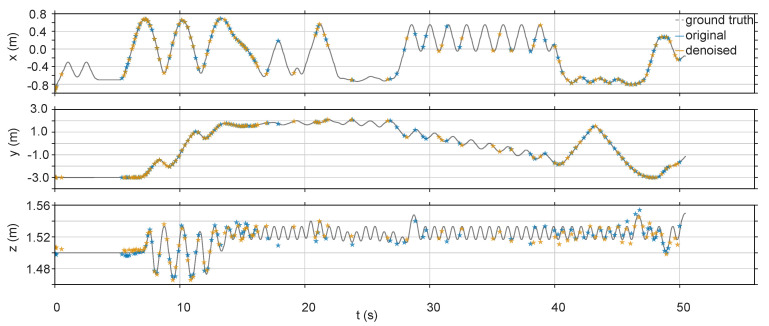
Comparison of the clean sequence, denoised clean sequence, and their ground truth on each coordinate axis.

The evo tools [39] are used to plot the figures of the estimated trajectories and the two trajectories with the RMSE closest to the average are selected for plotting. A visual comparison of the original and denoised sequence of the estimated trajectories are shown in Figure 8. The comparison on each axis of the clean sequence and denoised clean sequence is shown in Figure 9. Although the difference between the two is small, it can be seen from the z-axis that the denoised clean sequence error is smaller than the original clean sequence. The statistical results of Table 5 show that for the denoised sequences, the maximum of ATE decreased by 10.68%, the mean of ATE decreased by 17.23%, the median of ATE decreased by 19.55%, the minimum of ATE decreased by 6.35%, and the RMSE of ATE decreased by 16.75%.

To analyze whether the denoised sequences perform better than the clean sequence, the original clean sequence and the denoised sequence are evaluated for 20 times, the denoised sequence performs better on 15 occasions and the original clean sequence performs better on 5 occasions.

The probability *P* of obtaining *k* original sequences (perform better) in *n* tests with a probability of original sequences (perform better) equal to *p* is given by the binomial distribution:

(11) $$P\left(x=k\right)=\left(\begin{array}{c} n\\ k \end{array}\right)p^{k}{\left(1-p\right)}^{n-k}.$$

Then it can be checked whether the original sequences perform better than the denoised sequences:

Null hypothesis ($H_{0}$): The original sequences perform better than the denoised sequences, with probability *p* = 0.5.

Test statistic: Number of original sequences (perform better).

Alpha level (designated threshold of significance): 0.05.

Observation O: 5 original sequences (perform better) out of 20 experiments.

Left-tailed *p*-value of observation O given H0 is

$P=\sum_{i=0}^{5}\left(\begin{array}{c} 20\\ i \end{array}\right)0.5^{5}{\left(1-0.5\right)}^{20-5}=0.0207$.

The null hypothesis is rejected at the 0.05 level. Hence, this result (2.07% < 5%) indicates that we have evidence that is significant enough to reject the null hypothesis. So it can be said that the denoised sequence performed better than the original sequence.

#### 3.2.3. KITTI Sequences

KITTI dataset [18,19] is a combined dataset of images, lidar, GPS measurements, and inertial measurement unit accelerations recorded while driving near Karlsruhe, Germany, on a mobile platform.

Table 6 reports the results of the denoised sequences and the original sequence. However, only the RMSE after translation and scale alignment with the ground-truth trajectory is reported.

**Figure 10:**
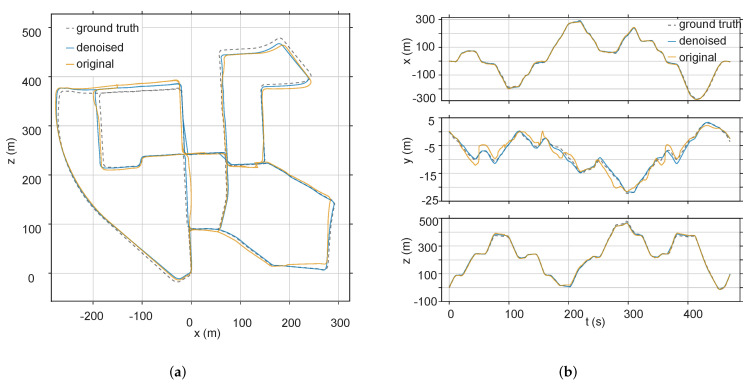
(**a**) Estimation statistics of the KITTI 00 sequence. (**b**) Comparison of the KITTI 00 sequence and its denoised sequence with ground truth on each coordinate axis.

The typical visual comparison between the original sequence and the denoised sequence (KITTI 00) is shown in Figure 10a, and the typical visual comparison on each axis of the original sequence and denoised sequence (KITTI 00) is shown in Figure 10b.

The 10 sequences on KITTI dataset are evaluated 10 times, and the results are weighted according to the number of frames. In 100 evaluations, the denoised sequences perform better on 64 occasions and the original sequences perform better on 36 occasions.

Similarly, it can be checked whether the original sequences perform better than the denoised sequences:

Null hypothesis ($H_{0}$): The original sequences perform better than the denoised sequences, with probability *p* = 0.5.

Test statistic: Number of original sequences (perform better).

Alpha level (designated threshold of significance): 0.05.

Observation O: 36 original sequences (perform better) out of 100 experiments.

Left-tailed *p*-value of observation O given H0 is

$P=\sum_{i=0}^{36}\left(\begin{array}{c} 100\\ i \end{array}\right)0.5^{36}{\left(1-0.5\right)}^{100-36}=0.0033$.

The null hypothesis is rejected at the 0.05 level. Hence, this result (0.33% < 5%) indicates that we have evidence that is significant enough to reject the null hypothesis, and it can be said that the denoised sequences performed better than the original sequences.

### 3.3. Feature Stability

Feature matching is at the base of many computer vision problems. In feature-based SLAM, when the matching accuracy is higher, the tracking effect is better. To find the reason why the matching rate of the denoised sequences is higher, the descriptors of matching image pairs are analyzed. The features in the two images are matched by the distance between the descriptors, where SIFT and SURF use the Euclidean distance, ORB uses the Hamming distance. The closer the distance, the higher the similarity of features.

In the matching pairs, if the feature descriptors on the match are completely consistent, they are considered to be the same feature and have better stability. These features are called “stable features”. The greater the number of “stable features”, the better the stability of feature matching.

In this paper, the clean sequence and the ORB descriptor are used for testing. Statistical analysis is performed on the number of all 1514 adjacent image pairs. Among the 1514 matched pairs, the average number of “stable features” in the denoised sequence is 354.02, where the number of the original clean sequence is 346.94. After the denoising, there are 1156 pairs have more number of stable feature, 302 pairs have less number stable feature and other 56 pairs have the same number of “stable features”.

**Figure 11:**
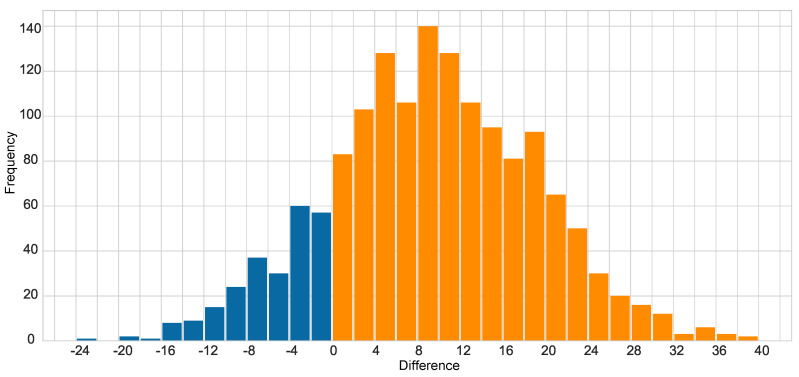
Frequency histogram of the difference between the “stable features” of the denoised and original clean pairs.

The difference between the “stable features” of the denoised pairs and the original clean pairs is also counted, the results are shown in Figure 11.

It can be seen from Figure 11 that the denoised pairs usually have 4–20 more “stable features” than the original clean pairs. Similarly, it can be checked whether the original pairs perform better than the denoised sequences:

Null hypothesis ($H_{0}$): The original clean pairs perform better than the denoised pairs, with probability *p* = 0.5.

Test statistic: Number of original pairs (perform better).

Alpha level (designated threshold of significance): 0.05.

Observation O: 302 original pairs (perform better) out of 1458 experiments.

Left-tailed *p*-value of observation O given H0 is

$P=\sum_{i=0}^{302}\left(\begin{array}{c} 1458\\ i \end{array}\right)0.5^{302}{\left(1-0.5\right)}^{1458-302}=4.55\times 10^{-118}$.

The null hypothesis is rejected at the 0.05 level. Hence, this result ($4.55\times 10^{-118}$ < 5%) indicates that we have evidence that is significant enough to reject the null hypothesis, and it can be said that the features in the denoised pairs are more stable.

**Figure 12:**
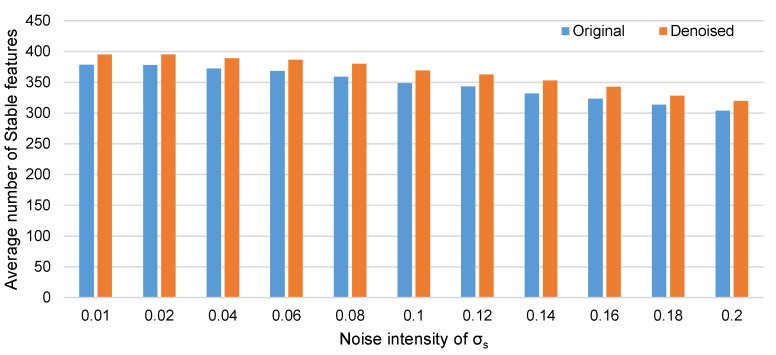
Stable features in different noise levels.

When the images to be co-registered, the noise in images often have different type and level. To study whether the denoised sequence has more stable features when the noise intensity is different, the original image and the denoised image in the clean sequence are used as a reference, and then images with different noise levels are performed image matching with the reference image respectively. The number of stable features is shown in Figure 12. It is shown that the denoised images have more stable features than the original. To some extent, it can be said that the denoising will be helpful when the image co-registration at different noise levels.

## 4. Conclusions

(1) This paper presents an open-source synthetic dataset to evaluate the noise of the visual SLAM methods by extending the WHU-RSVI dataset. The dataset contains 33 noisy sequences with various noise levels and the method to add image noise with customized noise types and levels is open-sourced.

(2) This paper uses FFDNet to denoise images in the dataset, and then SIFT, SURF, and ORB are employed for feature extraction and matching respectively. ORB has the highest matching rate at all noise levels, followed by SURF, and finally SIFT. At all noise levels, the denoised images always have a higher matching rate than the original image.

(3) This paper evaluates the performance of the noisy sequences and the denoised sequences under the ORB-SLAM2. The results show that the denoised sequences have a smaller ATE than the original image sequences. For the irradiance-independent Gaussian noise, the ATE of the denoised sequences is basically unaffected, while for the irradiance-dependent noise and mixed noise, the ATE of the denoised sequences decreases slightly. The denoised sequences are more robust than the original sequences.

(4) The clean sequence in the dataset is denoised by the FFDNet and the evaluated by ORB-SLAM2. Compared with the original sequence, the RMSE of ATE of the denoised clean sequence has decreased by 16.75%, indicating that when the features are sufficient, denoising the clean sequence can also improve the performance of the current monocular feature-based visual SLAM methods.

(5) The commonly used KITTI dataset sequences are evaluated, and compared with the original sequences, 7 out of 10 sequences have lower RMSE than the original sequences.

For an image without any noise, denoising the image will lose some information. However, it is found that after image denoising, the tracking accuracy of the ORB-SLAM2 is improved regardless of whether there is noise in the image or not. According to the analysis in Section 3.3, this is because the information affecting the feature descriptor is removed, so that the feature is usually more stable after denoising.

The work of this paper is low coupling with the existing SLAM methods, and it can be easily applied to existing methods. Moreover, using other denoising methods maybe also useful (We use DnCNN to denoise the clean sequence, and then use ORB-SLAM2 to evaluate it. Of the 20 evaluations, 16 of the evaluation results are better than the original sequence (*p*-value: 0.59% < 5%). The RMSE is 0.00660 m, an average drop of 32.83%). The image denoising in this paper belongs to the image pre-processing in the SLAM system, which has a similar effect to image enhancement. Therefore, an image denoising module can be added between the image capture module and feature tracking module when the engineers are developing relevant projects. Generally, FFDNet needs extra time to denoise images, but in offline feature-based SLAM, using FFDNet to denoise image sequences can improve the accuracy of the system. In structure from motion, the accuracy of the system is usually more important than the running time, so the researchers can also use FFDNet to pre-process the image first.

In future work, we will convert FFDNet’s PyTorch model to TorchScript and run it in C++, then FFDNet can be integrated with ORB-SLAM2 and the images denoised by FFDNet are input into ORB-SLAM2 for feature extraction and matching. Visual SLAM is a computationally intensive task, it is difficult for visual SLAM to achieve real-time performance in a resource-limited environment like micro aerial vehicles and mobile robots. So we plan to build a cloud computing service platform, the images captured by the camera will immediately transfer to the platform, then the image denoising, feature matching, bundle adjustment, loop detection, and other computationally intensive tasks can be performed in the cloud computing service platform. Besides, we will study the performance of the method under fast motions with motion blur. The noise dataset in this paper is for monocular, and the binocular and RGB-D sequences considered to expand.

## Figures and Tables

**Table 1:** Statistical information of the trajectories.

Frames	Duration	Length	Avg. Vel.
2895	96.50 s	50.11 m	0.52 m/s

**Table 2:** The average Peak Signal to Noise Ratio (PSNR) values (dB) of the noisy sequences and the denoised sequences.

Noise Level	Noisy	Denoised	Noise Level	Noisy	Denoised	Noise Level	Noisy	Denoised
($\sigma_{c},\sigma_{s}$)	Sequence	Sequence	($\sigma_{c},\sigma_{s}$)	Sequence	Sequence	($\sigma_{c},\sigma_{s}$)	Sequence	Sequence
(0.005, 0.0)	45.26	31.773	(0.0, 0.01)	43.35	31.771	(0.005, 0.01)	41.491	31.77
(0.01, 0.0)	39.835	31.769	(0.0, 0.02)	37.712	31.765	(0.01, 0.02)	35.717	31.761
(0.02, 0.0)	33.983	31.758	(0.0, 0.04)	31.804	31.753	(0.02, 0.04)	29.779	31.748
(0.03, 0.0)	30.500	31.75	(0.0, 0.06)	28.313	31.748	(0.03, 0.06)	26.277	31.751
(0.04, 0.0)	28.020	31.748	(0.0, 0.08)	25.838	31.75	(0.04, 0.08)	23.803	31.761
(0.05, 0.0)	26.097	31.753	(0.0, 0.10)	23.924	31.745	(0.05, 0.10)	21.895	31.739
(0.06, 0.0)	24.528	31.765	(0.0, 0.12)	22.368	31.698	(0.06, 0.12)	20.35	31.234
(0.07, 0.0)	23.205	31.783	(0.0, 0.14)	21.06	31.283	(0.07, 0.14)	19.058	29.167
(0.08, 0.0)	22.064	31.803	(0.0, 0.16)	19.936	30.011	(0.08, 0.16)	17.955	26.029
(0.09, 0.0)	21.062	31.792	(0.0, 0.18)	18.953	27.96	(0.09, 0.18)	17.000	23.135
(0.10, 0.0)	20.172	31.602	(0.0, 0.20)	18.083	25.621	(0.10, 0.20)	16.164	20.918

**Table 3:** Statistics of feature matching results on Scale Invariant Feature Transform (SIFT), Speeded Up Robust Features (SURF), and Oriented FAST and Rotated BRIEF (ORB).

		Original	Denoised
SIFT	N Points	990201	990225
	Matching Rate	00.4174	0.5016
SURF	N Points	989880	989825
	Matching Rate	0.6144	0.6424
ORB	N Points	990000	990000
	Matching Rate	0.7194	0.7399

**Table 4:** The Root-Mean-Square Error (RMSE) of Absolute Trajectory Error (ATE) of noisy sequences and denoised sequences at different noise levels (“×” means the tracking is lost).

Noise Level	Noisy	Denoised	Noise Level	Noisy	Denoised	Noise Level	Noisy	Denoised
($\sigma_{c},\sigma_{s}$)	Sequence	Sequence	($\sigma_{c},\sigma_{s}$)	Sequence	Sequence	($\sigma_{c},\sigma_{s}$)	Sequence	Sequence
(0.005, 0.0)	**0.00666**	0.00670	(0.0, 0.01)	0.00728	**0.00638**	(0.005, 0.01)	0.00705	**0.00632**
(0.01, 0.0)	0.00699	**0.00642**	(0.0, 0.02)	0.00722	**0.00648**	(0.01, 0.02)	0.00734	**0.00731**
(0.02, 0.0)	0.00741	**0.00690**	(0.0, 0.04)	0.00764	**0.00628**	(0.02, 0.04)	0.0148	**0.00652**
(0.03, 0.0)	0.01499	**0.00668**	(0.0, 0.06)	0.02285	**0.00669**	(0.03, 0.06)	0.0113	**0.00685**
(0.04, 0.0)	0.00925	**0.00698**	(0.0, 0.08)	0.02752	**0.00724**	(0.04, 0.08)	×	**0.00683**
(0.05, 0.0)	0.02619	**0.00654**	(0.0, 0.10)	×	**0.00678**	(0.05, 0.10)	×	**0.00665**
(0.06, 0.0)	×	**0.00669**	(0.0, 0.12)	×	**0.00657**	(0.06, 0.12)	×	**0.00743**
(0.07, 0.0)	×	**0.00664**	(0.0, 0.14)	×	**0.00816**	(0.07, 0.14)	×	**0.00801**
(0.08, 0.0)	×	**0.00672**	(0.0, 0.16)	×	**0.00788**	(0.08, 0.16)	×	×
(0.09, 0.0)	×	**0.00675**	(0.0, 0.18)	×	**0.00867**	(0.09, 0.18)	×	×
(0.10, 0.0)	×	**0.00689**	(0.0, 0.20)	×	×	(0.10, 0.20)	×	×

**Table 5:** Estimation statistics of the clean sequence.

Trajectories	Max Err (m)	Mean Err (m)	Median Err (m)	Min Err (m)	Rmse Err (m)	Std Err (m)
clean sequence ATE	0.022381	0.008707	0.00847	0.001498	0.00983	0.00453
denoised sequence ATE	0.01999	0.007207	0.006814	0.001403	0.008184	0.003854

**Table 6:** RMSE of ATE in meters after translation and scale alignment on the KITTI dataset (average over ten times).

Sequences	KITTI-00	KITTI-01	KITTI-02	KITTI-03	KITTI-04
Denoised	**6.218838**	**367.9401**	**22.4678**	1.758998	**0.841975**
Original	8.0375	375.0625	25.23833	1.171983	0.906354
Sequences	KITTI-05	KITTI-06	KITTI-07	KITTI-08	KITTI-09
Denoised	**5.746126**	16.42175	2.515805	**49.98243**	**42.10563**
Original	5.847668	14.66747	2.451807	51.24599	43.4026

## Abbreviations

The following abbreviations are used in this manuscript:

ATE	Absolute Trajectory Error
BN	Batch Normalization
CNN	Convolutional Neural Network
CRF	Camera Response Function
DnCNN	Denoising Convolutional Neural Network
DSO	Direct Sparse Odometry
FAST	Features from Accelerated Segment Test
FFDNet	Fast and Flexible Denoising convolutional neural Network
LSD-SLAM	Large-Scale Direct monocular SLAM
ORB	Oriented FAST and Rotated BRIEF
ORB-SLAM	ORB feature-based SLAM
POV-Ray	Persistence Of Vision Raytracer
PSNR	Peak Signal to Noise Ratio
RANSAC	RANdom SAmple Consensus
ReLU	Rectified Linear Unit
RGB-D	Red Green Blue-Depth
RMSE	Root Mean Square Error
SIFT	Scale Invariant Feature Transform
SLAM	Simultaneous Localization and Mapping
SURF	Speeded Up Robust Features
SVO	Semidirect Visual Odometry
